# Pupil dynamics after in-the-bag versus anterior and retropupillary iris-fixated intraocular lens implantation

**DOI:** 10.1038/s41598-021-01051-6

**Published:** 2021-11-02

**Authors:** Yanxiu Sun, Maximilian Hammer, Timur M. Yildirim, Ramin Khoramnia, Gerd U. Auffarth

**Affiliations:** 1 Department of Ophthalmology, Peking University Third Hospital, Northgarden Road 49, Haidian District, Beijing, 100191 China; 2 International Vision Correction Research Centre (IVCRC), Department of Ophthalmology, Ruprecht-Karls University of Heidelberg, Im Neuenheimer Feld 400, 69120 Heidelberg, Germany

**Keywords:** Lens diseases, Eye diseases, Pupil disorders

## Abstract

An Intraocular Lens (IOL) fixated on the iris either anteriorly, as a phakic IOL, or posteriorly, as an aphakic IOL, can influence pupil motility. In this interventional case series study, we evaluated pupil size under different levels of illumination (scotopic = 0.04 lx, low-mesopic = 0.4 lx and high-mesopic = 4 lx) for anterior iris-claw IOL fixation for correcting myopia or hyperopia (IFPH), retropupillary iris-claw IOL fixation to correct aphakia or as treatment for late in-the-bag IOL dislocation/subluxation (IFRP), and capsular-fixation IOL in-the-bag implantation (IB). Pupil size was measured preoperatively for the IFPH- and IB-group as well as 6 months after surgery for all groups. We analyzed a total of 70 eyes: 22 eyes of 11 patients with phakic IOLs, 22 eyes of 20 patients in the IFRP group and 26 eyes of 13 patients in the IB group. Both IFPH and IB showed a smaller postoperative scotopic pupil size, compared with the preoperative values. When compared to postoperative values of IB and IFPH, IFRP showed a significantly smaller postoperative scotopic pupil size (IFPH: 5.89 ± 0.83 mm, IFRP: 4.37 ± 0.83 mm, IB: 5.34 ± 0.98 mm, p < 0.001) while no differences were seen at high-mesopic lighting. Neither of the surgical techniques seems to impair the constriction of the pupil.

## Introduction

Iris-fixated intraocular lenses (IOLs) are currently used for two indications. The anterior fixation is used for correcting phakic patient’s refractive errors and therefore competes with other methods such as the phakic posterior IOL implantation and refractive lens replacement. The second implication is to treat aphakia or the late in-the-bag IOL dislocation/subluxation. Even though the iris-claw IOL was designed for fixation on the anterior iris surface, in case of aphakia correction, it is preferred to fixate the IOL behind the iris to spare the anterior chamber morphology and reduce corneal endothelium damage during intraocular manipulation of the IOL^1^. Next to transscleral IOL fixation, which holds inherent risks like ciliary body haemorrhage^2–7^, iris fixation is used to overcome the challenge of insufficient capsular support, especially in Europe and Asia^8–10^. Helvati et al. showed that visual acuity does not differ between anterior and retropupillary placement of the iris-claw lens for treating aphakia^11^. There is substantial evidence on the favourable short- and long-term outcomes of iris-claw IOLs in the treatment of IOL dislocation qualifying it as a valid alternative to scleral fixated or angle-supported IOLs. It is an efficient and safe surgical procedure^12–15^.

Regardless of the placement of the iris-claw IOL, the enclavation of the iris increases the risk of inflammatory reactions that might escalate to complications such as anterior uveitis^9^. Additionally, iris-fixation might restrict pupil motility: a dilated or distorted pupil might cause photophobia; persistent pupil constriction can impede clinical examination of the posterior segment, which is especially undesirable in cases with additional vitreoretinal disorders.

In this study, we sought to investigate the postoperative pupil size at three different levels of illumination. We compared three surgical strategies of IOL fixation: anterior Iris-fixation in phakic eyes to treat refractive errors (**I**ris-**f**ixated **ph**akic = IFPH group), retropupillary iris-fixation (**I**ris-**f**ixated **r**etro**p**upillary = IFRP group) and capsular IOL implantation **i**n-the-**b**ag (IB group) to treat aphakia. Additionally, pre- and postoperative pupil size was compared for the IFPH and the IB group.

## Material and methods

### Patient selection

This was a non-randomized interventional case series study. Patients were recruited at the Department of Ophthalmology, Heidelberg University Hospital, Heidelberg, Germany. This study was approved by the ethical committee of Heidelberg University (S-392/2011). All patients gave written informed consent to participation in this study. This study was conducted in accordance with internationally recognized guidelines, including Good Clinical Practice (ICH-GCP) and the Declaration of Helsinki.

We included patients undergoing (1) correction of myopia or hyperopia with phakic anterior chamber iris-claw IOL fixation (IFPH) (2) correction of aphakia with retropupillary iris-claw IOL fixation (IFRP) as well as (3) patients with capsular-fixation IOL in-the-bag implantation (IB). Patients were excluded if preoperative examination showed iris defects or possible pupil motility disorders due to parasympathetic medication or other systemic diseases, such as diabetes, if anterior or posterior synechiae were observed or if no sufficient intraoperative pupil dilation was achieved.

### Measurements

Pupil size was measured pre- and postoperatively for the IFPH and IB-group. Due to the possible impairment of pupil dynamics prior IFRP surgeries due to a dislocated IOL or a bulbus trauma, no preoperative values were obtained for this group. Pupil size was measured 6 months after surgery in all groups to minimize immediate postoperative bias.

The pupil size was measured using a Procyon P2000 pupillometer (Procyon Instruments Ltd, London, United Kingdom) at three levels of luminance: Scotopic at 0.04 lx; low-mesopic at 0.4 lx; high-mesopic at 4.0 lx.

### IOL choice

Phakic anterior chamber iris-claw IOL fixation for correcting myopia or hyperopia (IFPH) group: Different models of IOLs were used for correcting myopia by anterior iris-claw IOL fixation: Verisyse VRSM 50, VRSM 60, and VRSH 50 (Abbott Medical Optics, Santa Ana, California, USA), which all have a 5.0 mm optic and 8.5 mm overall diameter.

Retropupillary iris-claw IOL fixation to correct aphakia (IFRP) group: Verisyse VRSA 54 Aphakic Iris Claw IOL (Abbott Medical Optics, Santa Ana, California, USA), which has the same dimensions as the IFPH IOLs, was used for cases undergoing retropupillary fixation.

In-the-bag implantation (IB) group: M-flex 630F IOL (Rayner Intraocular Lenses Limited, Hove, East Sussex, UK) for correcting aphakia were used in the in-the-bag fixation group.

#### Retropupillary iris-claw fixation (IFRP)

Two paracenteses were made at 3 and 9 o’clock positions. A high viscosity Ophthalmic Viscosurgical Device (OVD), Healon GV (Abbott Medical Optics, Santa Ana, CA, USA) was injected into the anterior chamber through one paracentesis. A clear corneal incision was made at 12 o’clock. Either the remnants of the lens material or the dislocated IOL were removed followed by anterior vitrectomy. The IOL was inserted in the anterior chamber under OVD protection. Using a forceps, it was brought into position behind the pupil and fixated on the mid-peripheral iris, following the method described by Baykara et al.^16^ The OVD was removed and the main incision was sutured with 10-0 Nylon.

#### Regular in-the-bag implantation (IB)

Paracenteses and the main incision were similarly placed as in patients from the IFRP group. The capsulorhexis was performed and followed with hydrodissection, hydrodelineation and phacoemulsification. Throughout the surgery, bi-manual irrigation-aspiration was applied. The IOL was injected into the capsular bag. Finally, the OVD was removed, and the incisions were hydrated.

#### Phakic anterior chamber iris-claw fixation (IFPH)

Peripheral iridectomy using a YAG laser was performed before surgery. A 6 mm sclero-corneal or clear corneal incision was performed. Paracenteses were placed as mentioned for the IFRP and IB groups. The IOL was inserted into the anterior chamber and placed at the 3 to 9 o’clock position and the haptics were enclavated in the iris. OVD was removed and the incision was sutured with 10-0 Nylon.

### Statistical analysis

Kolmogorov–Smirnov test was used to test normal distribution within each surgery group at every level of illumination. In order to account for data correlation on multiple levels (the eye level and the patient level), linear mixed effect models were used to statistically analyze the data. In case of comparing pre-and postoperative values as well as comparing data from the same eye at different levels of illumination, we used a two-level mixed effect model. For comparison between surgical groups, one-level mixed effect models were used. STATA 17 (StataCorp, LLC, Texas, USA) and PRISM 8 (GraphPad Software, LLC) were used for statistical analysis.

### A priori sample size calculation

An a priori sample size estimation was conducted. Considering a difference of 1 mm between groups with a standard deviation of 1 mm at scotopic illumination as a clinically relevant change in pupil dynamics, an effect size of 1 (Cohen’s d) can be assumed. To detect a significant difference between the mean of two independent groups, in this case types of surgery, a sample size of 18 observations per group is needed to achieve a power of 90%. The only available study that dealt with a similar research question is Dick et al.^17^ Dick et al. examined 22 myopic eyes that underwent anterior phakic iris-claw IOL-implantation. Thus, we decided to include a minimum of 22 eyes per group.

## Results

A total of 70 eyes were analyzed in this study: 22 eyes of 11 patients in the IFPH group, 22 eyes of 20 patients in the IFRP group and 26 eyes of 13 patients in the IB group. Preoperative data was available for 20 (90.9%) and 18 (69.2%) eyes in the IFPH-group and IB-group, respectively. As previously mentioned, no preoperative data was gathered for the IFRP group due to the possible influence of the dislocated IOL or the bulbus trauma on pupil dynamics.

The mean age of patients was 34, 63 and 68 years for the IFPH, IFRP and IB-group, respectively. 36%, 73% and 46% of the patients were male for the IFPH, IFRP and IB-group respectively. Table 1 presents the background demographics of the studied patient population.

**Table 1:** Background demographics of the study population.

	IFPH	IB	IFRP
Number of subjects	11	13	20
Number of eyes	22	26	22
Age (mean ± SD)	34 ± 8	68 ± 8	63 ± 14
Male sex	4 (36.4%)	6 (46.2%)	16 (80%)
Diabetes	0	0	3 (15%)
PEX	0	0	2 (9.1%)
Indication for surgery	–	–	Aphakia after complications during crystalline lens removal—11 (50%)
			IOL subluxation—7 (31.8%)
			Trauma—2 (9.1%)
			PEX with no IB possible—2 (9.1%)

In the IFPH group the two main reasons for implanting an iris-fixated retropupillary lens were: (1) to treat aphakia after complications during crystalline lens removal (11 eyes, 50%) and (2) to treat aphakia after complicated IOL subluxations (7 eyes, 31.8%). Two patients suffered a trauma of the crystalline lens and capsular bag. Finally, the 2 remaining patients showed signs of pseudoexfoliation-syndrom (PEX) with no in-the-bag implantation possible.

### Comparison of pre- and postoperative pupil size

**Figure 1:**
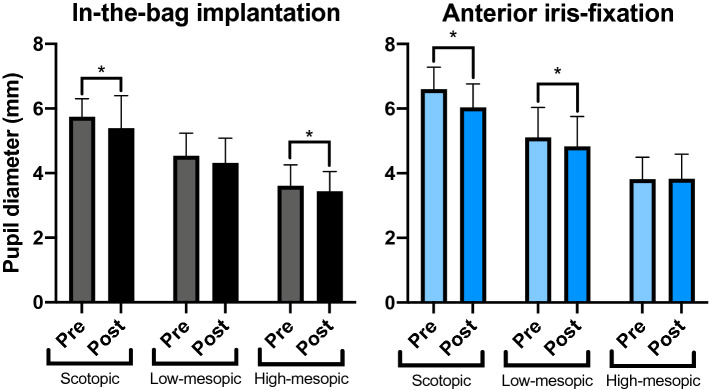
Preoperative versus postoperative pupil size for anterior iris fixation and in-the-bag implantation at different levels of illumination Preoperative data was available for a subset of patients undergoing IB and IFPH. After surgery, both groups showed significantly decreased maximum pupil size under scotopic lighting compared to preoperative values.

Figure 1 compares pre- and postoperative pupil sizes at different levels of illumination for both groups with available preoperative data, namely IFPH and IB. Both groups showed a reduced pupil size under scotopic illumination postoperatively (p < 0.001 and p = 0.03, two-level linear mixed effect model for IFPH and IB, respectively). Table 2 presents mean pre- and postoperative values. Additionally, a significantly smaller postoperative pupil size was seen for the IFRP-group at the low-mesopic lighting (p = 0.02) and for the IB-group at high-mesopic lighting (p = 0.03).

**Table 2:** Pre- and postoperative pupil dynamics in patients undergoing regular in-the-bag IOL implantation and phakic Iris-Claw IOL implantation.

	Scotopic	Low-mesopic	High-mesopic
**In-the-bag IOL implantation**			
Preoperative pupil diameter (mm)	5.74 ± 0.56	4.54 ± 0.70	3.61 ± 0.65
Postoperative pupil diameter (mm)	5.39 ± 1.01	4.31 ± 0.77	3.44 ± 0.61
P value	0.031*	0.056	0.034*
**Phakic Iris-Claw IOL implantation**			
Preoperative pupil diameter (mm)	6.61 ± 0.68	5.11 ± 0.93	3.82 ± 0.67
Postoperative pupil diameter (mm)	6.04 ± 0.73	4.83 ± 0.93	3.83 ± 0.76
P value	< 0.001*	0.024*	0.895

P-values origin from a two-level linear mixed model. *indicates statistical significance with p < 0.05.

### The effect of illumination on postoperative pupil size

**Figure 2:**
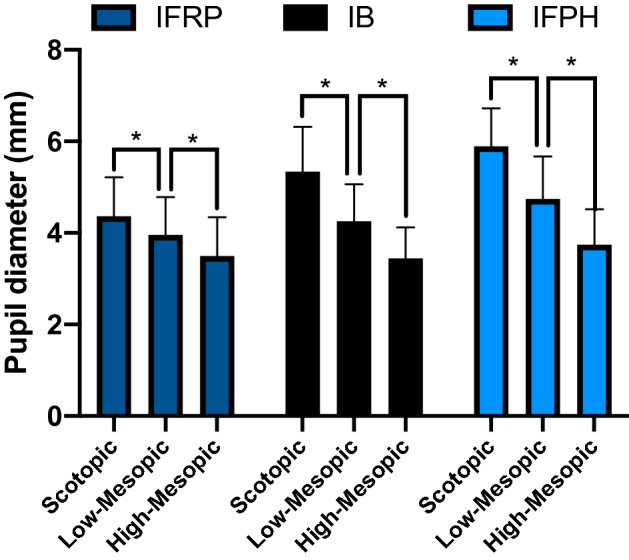
The effect of illumination on postoperative pupil size. Statistically significant changes in pupil diameter were observed between the 3 levels of illumination within each surgical group.

Increasing illumination resulted in significantly smaller pupil size in all groups (Fig. 2, p < 0.001 for all, two-level linear mixed effect models). Table 3 presents the pupil size in mm for the 3 different levels of illumination.

**Table 3:** Mean pupil size of patients after IFRP, IB and IFPH under 3 different levels of illumination.

	Pupil diameter (mm)		
	Scotopic	Mesopic low	Mesopic high
IFRP	4.36 ± 0.85	3.96 ± 0.83	3.50 ± 0.85
IB	5.34 ± 0.98	4.26 ± 0.80	3.44 ± 0.68
IFPH	5.89 ± 0.83	4.74 ± 0.92	3.74 ± 0.77

### Comparison of pupil size between types of surgeries

**Figure 3:**
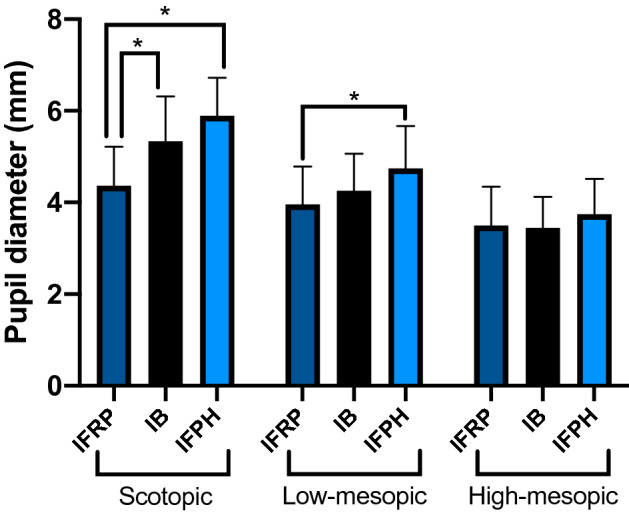
Comparison of pupil size 6 months after surgery at different levels of illumination for IFRP, IB and IFPH. Under high-mesopic lighting, no difference in pupil diameter occurred. IFRP showed a decreased pupil diameter for low-mesopic and scotopic lighting. IFPH showed slightly greater pupil size compared to IB.

Figure 3 depicts the statistical comparison between pupil size and type of surgery by the level of illumination (one-level linear mixed effect model) Pupil size was not significantly different between groups at high-mesopic illumination (p = 0.46 for IFRP vs IFPH, p = 0.78 for IFRP vs IB, p = 0.12 for IB vs IFPH, respectively). At low-mesopic illumination, IFRP showed a significantly smaller pupil size than IFPH (p = 0.02). At scotopic illumination, IFRP showed a statistically smaller pupil size than the IB and IFPH-group (p < 0.0001 for IFPH vs IFRP, p = 0.12 for IFPH vs IB, p = 0.002 for IFRP vs IB).

### Sensitivity analysis

A total of 4 patients that underwent IFRP showed signs of PEX or a traumatic cataract. To address these possible confounding factors of scotopic pupil size and motility, we excluded these patients in a sensitivity analysis and analyzed differences in pupil size. Still, no significant difference was observed at high-mesopic illumination (p = 0.60 for IFRP vs IB, p = 0.10 for IFRP vs IFPH), whereas a smaller scotopic pupil size was observed for IFRP compared to IB and IFPH (p < 0.0001 for IFRP vs IB, p < 0.0001 for IFRP vs IFPH).

## Discussion

We compared pupil diameter of 3 surgical techniques at 3 different levels of illumination. In eyes undergoing IFPH and IB, we noticed a significant decrease of pupil diameter at scotopic illumination after surgery. Additionally, 6 months after surgery we observed a decreased pupil diameter after IFRP, when compared to IFPH or IB. Neither of the surgical techniques seems to impair the constriction of the pupil. To our knowledge, this study is the first to systematically assess postoperative pupil size in patients undergoing retropupillary iris-fixated IOL implantation to treat aphakia.

Pupil size influences the visual outcome after IOL implantation. If the pupil size exceeds the IOLs’ optical zone diameter after IFPH, higher-order aberrations could be markedly increased^18^. Glare phenomena and poor contrast sensitivity are frequently observed postoperatively under scotopic conditions^19^. An adequate prediction of postoperative pupil size is needed to determine, which patients benefit from IFPH.

Only few studies discuss changes of pupil size after cataract surgery. Some either examined special patient populations such as diabetics^20^, other clinical conditions, such as posterior chamber phakic IOLs^21^ or do not present longitudinal data^22^. When Jan Worst and associates introduced anterior iris-claw IOL surgery, reported in 1972, no changes in pupil motility or diameter were reported^23^. Normal iris physiology including vascularity and innervation were observed^23^. In this study, we report a significant decrease in pupil diameter under scotopic illumination in the IFPH group after surgery when compared to preoperative values. This result is in line with data presented by Dick et al.^17^ In this study, Dick et al. used a similar follow-up time frame of 6 months. They noted a decrease of pupil diameter under scotopic conditions of roughly 1 mm for both, myopic und hyperopic eyes. In our study, we observed a decrease of scotopic pupil diameter of 0.6 mm in the IFPH group. In both studies, the mean age of patients was comparable and represents the patient population undergoing IFPH: In our study, the mean age was 35. Decreased pupil size might be explained by the enclavation of the iris tissue, limiting pupil motility. After the implantation of an Iris-fixated lens, a chronic traction of the haptic at the midperipheral iris is induced, that may lead to subclinical inflammation at 1 and 2 years after surgery^24^. This could lead to pupil alterations and could explain the smaller scotopic pupil diameter^25,26^.

However, Koch et al. observed changes in pupil size after phacoemulsification itself, regardless of the implanted IOL offering an alternative explanation not related to a mechanical impairment because of the enclavation of the iris. This is in line with our results in the IB-group. Here we also found a significant decrease in scotopic pupil size after surgery. Two studies were previously conducted in patients undergoing IB-implantation by Sobaci et al. and Hayashi et al. Hayashi et al. assessed pupil size 3 days and 1 month after surgery. They saw a similar decrease of scotopic pupil size of 0.5 mm in non-diabetic patients after 3 days as we observed after 6 months, however only 0.2 mm at 1 month postoperatively. The mean age of our IB-patient cohort was comparable to the cohort of Hayashi (68 vs 69 years, respectively). Kanellopoulos et al. also investigated pupil size after IB-implantation in 86 eyes with a mean age of 71 years. At 3 months after surgery, they still noted significant decreases in pupil size of 0.3 mm based on measurement with Scheimpflug Imaging. Interestingly, they found an aggravation of changes in pupil size with increasing age. They also present another possible mechanism for the changes in pupil size: The increase of anterior chamber depth. The increased freedom of movement of the iris after surgery may lead to a reduced pupil diameter.

We evaluated the postoperative pupil size of IFPH, IB and IFRP at three different levels of illumination. Age is an important factor to be considered when comparing pupil size after surgery between groups^27,28^. The mean age of patients was 35, 63 and 68 years for the IFPH, IFRP and IB-group, respectively. Based on age, the IFRP and IB-group are within a similar age range and therefore should be comparable. However, the IFPH-group is much younger than both other groups. Thus, the comparison of pupil dynamics between all three groups may be influenced by the difference in age. A recent study of Fernández et al. researched the pupil diameter in patients with multifocal IOLs under conditions that are similar to our low-mesopic measurements. The group of 61-to-70 years old patients had a mean pupil diameter of 4.13 mm at low-mesopic lighting. The pupil diameter of the IB group was in line with this, at low-mesopic lighting, with 4.31 mm. Compared to the IB-group, patients undergoing the IFRP-procedure showed a significantly smaller scotopic pupil size. Iris atrophy and chronic inflammation after complicated cataract surgery or previous trauma of the iris, such as subluxations of the crystalline lens, may induce pupil deformations and therefore impair dilation^25,26,29^. Pérez-Santonja et al. reported, that inflammation is still present one year after iris fixation surgery^24^. Next to patients’ age, other parameters can influence pupil dynamics irrespective of the type of surgery performed, such as PEX. In the IFRP group, 18 of 22 underwent IFRP to either treat IOL-subluxations or as a result of a loss of capsular support during a previous cataract surgery. Only 2 patients showed signs of PEX, 2 patients underwent IFRP due to a traumatic cataract. Consequently, to address this concern, we excluded the latter 4 patients with possible confounding factors as a sensitivity analysis. All results remained robust.

Interestingly, the high-mesopic pupil size at near maximum pupil constriction did not differ between the groups. This is once again in line with data presented by Dick et al. as well as Fernandéz et al.

This change of pupil dynamics in the IFRP group could cause various impairments after surgery: The reduced pupil diameter under scotopic illumination in the IFRP group might impair night vision, as the passage of light through the smaller diameter will be limited. On the other hand, postoperative complications such as glare and high order aberrations which are more present in individuals with larger pupils^30^ could be reduced or even prevented through smaller pupil sizes. In addition, a smaller pupil can increase the depth of focus which will one the one hand counteract some of the variation of IOL power calculation and might give an increase in depth of vision^31^.

Our study has limitations: While preoperative data was available for IFPH and IB, no data could be acquired for the IFRP group due to the possible influence of a trauma or the dislocated IOL on the pupil dynamics before surgery. However, the age of the IB-group was comparable to the IFRP-group and only showed a difference at scotopic lighting while showing similar results at high- and low-mesopic lighting. Additionally, we used one of many methods to determine pupil size. Different types of pupil measurements lead to different results^32,33^. This needs to be considered by the treating surgeon.

In conclusion, IFPH and IB showed decreased scotopic pupil size when compared to preoperative values. IFRP showed a statistically significant smaller pupil size at scotopic lighting when compared to the IB-group with a similar age. Neither of the surgical techniques seems to impair the constriction of the pupil. Further studies should assess pre- and postoperative pupil size of patients undergoing IFRP as well as the relation between pupil dynamic measurements and patient reported outcomes.

## Data Availability

Data is available upon reasonable request from Gerd Uwe Auffarth.
